# α-Therapy and Combination Strategies to Overcome Resistance and Enhance Clinical Outcomes in Prostate Cancer

**DOI:** 10.2967/jnumed.125.270940

**Published:** 2025-12

**Authors:** Anna Karmann, Stephen Rose, Ken Herrmann, Clemens Kratochwil, Oliver Sartor, Shahneen Sandhu, Louise Emmett

**Affiliations:** 1 AdvanCell Pty Ltd., Cambridge, Massachusetts, and Sydney, New South Wales, Australia;; 2 Department of Nuclear Medicine, University of Duisburg-Essen, and German Cancer Consortium–University Hospital Essen, Essen, Germany;; 3 Department of Nuclear Medicine, University Hospital Heidelberg, Heidelberg, Germany;; 4 Transformational Prostate Cancer Research Center, LCMC Health, New Orleans, Louisiana;; 5 Division of Cancer Medicine, Peter MacCallum Cancer Centre, Melbourne, Victoria, Australia; and; 6 Department of Theranostics and Nuclear Medicine, St Vincent’s Hospital Sydney and St Vincent’s Clinical School, University of New South Wales, Sydney, New South Wales, Australia

More effective treatments for metastatic prostate cancer (mPC) are needed as disease progression remains universal. ^177^Lu-PSMA-617 radiopharmaceutical therapy (RPT)—Pluvicto (Novartis)—was first approved for the treatment of castration-resistant prostate cancer (PC) based on the landmark VISION trial (1). Although Pluvicto is the “poster child” of RPT, the median overall survival benefit is modest at 4 mo (1). Prostate-specific membrane antigen (PSMA)–targeting therapy with α-emitting payloads (α-PSMA) may advance the clinical benefit of RPT. The enhanced cytotoxicity of high-energy α-particles provides the potential for less upfront radiation resistance and superior antitumor activity compared with current approved β-emitters.

Nevertheless, the heterogeneity of PSMA expression within and among tumors presents a challenge for achieving durable responses, which may be potentially aggravated by the short pathlength of α-payloads. PSMA expression, encoded by the *FOLH1* gene, is heterogeneous across metastatic sites and molecular subtypes of mPC. Immunohistochemical studies have reported 98% of metastatic deposits comprise a mix of both PSMA-positive and PSMA-negative cells, in both metastatic hormone-sensitive PC (mHSPC) and metastatic castration-resistant PC (mCRPC) (2). Despite the expected improved efficacy of α-therapy compared with β-therapy, clonal heterogeneity and radiation resistance remain key causes of primary treatment resistance or early treatment failure of RPT. Herein, we discuss potential synergistic combination therapies with α-PSMA that may enhance the depth and durability of response in men with PC while limiting potential additional toxicities.

## α-EMITTING PAYLOADS: MECHANISM OF ACTION

**FIGURE 1.**
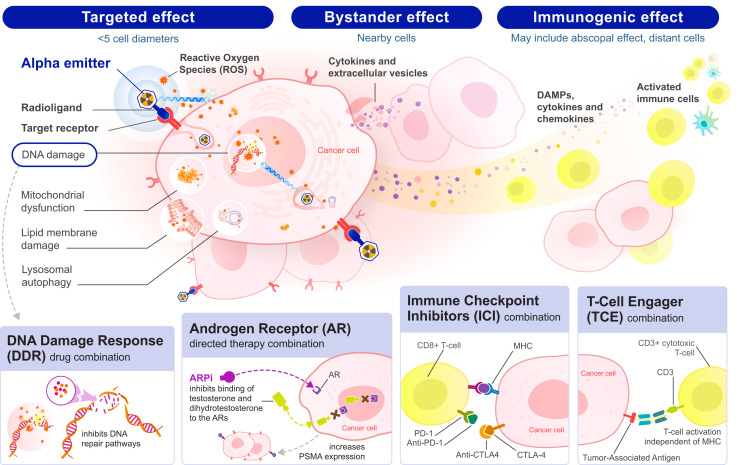
Mechanisms of action by PSMA-directed α-therapy including targeted effect, bystander effect, and immunogenic effect and potential synergies with other therapies enhancing tumor cell killing in PC. Synergetic combinations include DNA damage response pathway inhibitors, AR-directed therapy such as ARPI and AR degrader, ICIs, and T-cell engagers. DAMPs = damage-associated molecular patterns; MHC = major histocompatibility complex.

α-isotopes emit high-energy helium nuclei (5–8 MeV) with a linear energy transfer up to 100 keV/μm, deposited over a short range of approximately 3–4 cell diameters (25–80 μm), causing DNA damage irrespective of the cell cycle stage or oxygenation state of the tumor. Figure 1 shows the multiple potential mechanisms of action of α-emitters including the well-established DNA damage (both double- and single-strand breaks) by α-emission as well as subcellular structure effects and cell membrane damage caused by reactive oxygen species; bystander effects through chemical signaling, causing cytotoxic damage to nonirradiated cells in close proximity to irradiated cells; and potentially immunogenic cell death caused by CD8+ T-cell immune response through release of cytokines, chemokines, and damage-associated molecular patterns (3,4). α-emitting isotopes with a short half-life thereby have, in theory, decayed at the time of immune infiltration into the tumor environment, providing a potentially enhanced immunogenic profile. This may apply to ^212^Pb and ^211^At relative to longer-lived isotopes. The radioactive decay of short half-life α-emitters further aligns with the biologic half-life of most peptide-based PSMA radiopharmaceuticals, delivering a maximum dose during the peak therapeutic window. These characteristics may provide a favorable toxicity profile compared with longer half-life α-emitters with multiple daughters that have potential for more serious marrow and other off-target toxicities especially when chelated to antibodies (5). Additionally, concerns have been raised around the unbound daughter nuclides from ^225^Ac, in particular, ^213^Bi, which has a 46-min half-life and may accumulate in the kidneys, as well as ^221^Fr, which may accumulate in the salivary glands and may partially explain the excess salivary gland toxicity of ^225^Ac-PSMA (6,7).

## COMBINATION STRATEGIES WITH AR SIGNALING PATHWAYS

The value of combining androgen receptor pathway inhibitors (ARPIs) and PSMA-targeted therapy may be two-fold. Upregulation of PSMA expression in androgen-resistant clones improves cellular uptake of PSMA-directed therapy, thereby delivering a greater radiation payload, whereas the ARPI will also treat low PSMA-expressing clonal populations less likely to respond to PSMA RPT (8). This conceptual synergy has been established in the ENZA-p phase 2 clinical trial, in which enzalutamide plus ^177^Lu-PSMA-617 improved overall survival in patients with mCRPC, not previously treated with ARPI, compared with enzalutamide alone (34 mo vs. 26 mo, respectively) (8). Imaging studies have reported increased PSMA expression on PET in patients with mCRPC after treatment with enzalutamide (9–12). Although ARPIs increased PSMA expression in castration-resistant disease and showed added value with ^177^Lu-PSMA RPT, the magnitude and timing of ARPIs with an α-therapy insult, the benefit of a second-line ARPI, as well as the combination in hormone-sensitive disease are yet to be fully explored (10). Of note, hormonal therapies may also enhance radiation-induced cell death via non-PSMA–mediated mechanisms through potential radiation sensitizing effects (13). Androgen receptor (AR) degraders are in clinical development to potentially overcome resistance in androgen-driven PC cells. In contrast to inhibiting AR function, AR degraders cause the receptor to be destroyed within the cell and have the potential to also increase PSMA expression in mCRPC.

As most patients with mCRPC will have been exposed to ARPIs in the mHSPC setting and eventually develop resistance to ARPIs, a strategy of combining PSMA-directed α-therapy with an effective AR degrader could lead to improved responses compared with approaches using ARPI switch combinations.

## COMBINATION STRATEGIES ADDRESSING IMMUNE MODULATION

Although several immune checkpoint inhibitors (ICIs), including programmed cell death protein 1 (PD-1) and anti–cytotoxic T-lymphocyte-associated protein 4 (CTLA-4) agents, have shown limited single agent efficacy in mPC (14), a body of preclinical data supports the translation of RPT–ICI combination therapies to the clinic (15–17). In a single-arm study, a single priming dose of ^177^Lu-PSMA-617, followed by maintenance pembrolizumab, provided encouraging activity in patients with mCRPC (18). The PRINCE phase 1 clinical trial, combining ^177^Lu-PSMA-617 and pembrolizumab in patients with mCRPC, has demonstrated provocative activity (19). Further support comes from dual checkpoint blockade with CTLA-4 and PD-1 inhibitors in combination with ^177^Lu-PSMA-617 improving PSA progression-free survival and radiographic progression-free survival in the EVOLUTION phase 2 trial, though toxicity concerns were apparent (20). The high linear energy transfer of α-therapy and high dose rate potentially elicit a stronger immune response by generating more DNA damage and potentially higher levels of tumor neoantigen presentation that could activate critical pathways such as the STING pathway to augment antitumor activity in combination with ICIs (21). To leverage the clinical potential of these combinations, the dosing schedule of α-PSMA–ICI and patient selection in future trials may be key to demonstrate isotope immune interactions and optimize the risk–benefit for the individual.

Various heterodimeric T-cell engagers are currently being developed, inducing T-cell–mediated cancer cell killing by binding the CD3 receptor on T cells while also binding a specific antigen expressed on the surface of PC cells, such as DLL3, KLK2, or STEAP-1. Harnessing the potential synergy of isotopes and T-cell engagers presents an exciting opportunity to explore new therapeutic dual-target and dual-modality combinations for mPC. The safety profile of immune modulation combination approaches requires careful attention and risk–benefit assessment.

## COMBINATIONS WITH DNA DAMAGE REPAIR MECHANISMS

Recent insights into the biology of PC have shown that several patients harbor clinically actionable molecular aberrations (22). Of these, up to 20% of men with mCRPC have alterations in genes implicated in the homologous DNA damage repair pathway that could be targeted using a poly(adenosine diphosphate–ribose) polymerase inhibitor (PARPi) (22,23). PARPi can act as a radiosensitizer by blocking the repair of single-strand DNA breaks, leading to replication-associated double-stranded DNA damage and providing potential synergism with DNA-damaging therapies (24). Studies suggest that radiation resistance may contribute to cancer progression after ^177^Lu- and ^225^Ac-PSMA therapy, potentially through alterations in DNA damage repair pathways (25,26). Potential mechanisms to overcome this resistance with optimized dose and schedule of the DNA damage response repair pathway inhibitors in combination with the isotopic therapies warrant exploration.

## TRANSLATION INTO CLINICAL DEVELOPMENT

α-PSMA therapy provides unique opportunities for synergistic combinations with other modalities to enhance clinical outcomes in men with PC. Rapid translation into clinical development and prioritization for registrational precision trials are needed to advance the impact of this modality and ensure patient access to optimal combination therapies.

## DISCLOSURE

Anna Karmann has stock options with AdvanCell. Stephen Rose has stock options with AdvanCell. Ken Herrmann receives consultant fees from Advanced Accelerator Applications, a Novartis company, Actithera, Amgen, AstraZeneca, Bain Capital, Bayer, Boston Scientific, Convergent, Curium, Debiopharm, EcoR1, Fusion, GE HealthCare, Immedica, Isotopen Technologien München, Janssen, Merck, MSD, Molecular Partners, NVision, POINT Biopharma, Pentixapharm, Pfizer, Radiopharm Theranostics, Rhine Pharma, Siemens Healthineers, SOFIE Biosciences, Telix, Theragnostics, and Y-mAbs; receives research grants from Advanced Accelerator Applications, a Novartis company, Boston Scientific, and Janssen; and has stock or other ownership interests with AdvanCell, Aktis Oncology, Convergent, NVision, SOFIE Biosciences, and Yellowbird Diagnostics. Oliver Sartor receives consultant fees from AbbVie, Abdera, Actithera, AdvanCell, Alpha9, Amgen, ARTbio, Astellas, AstraZeneca, Bayer, Clarity Pharmaceuticals, Convergent, Curadh, Curium, Fusion, ITM Oncologics, JNJ, Lantheus, Merck, Norroy, NorthStar, Novartis, Nucleus Biopharma, Pfizer, RATIO, Sanofi, Swiss Rockets, Telix, and Zalpha; has stock options with Abdera, Actithera, AdvanCell, ArtBio, Clarity, Convergent, Ratio, and Telix; and receives research grants and other clinical trial support (to the institution) from Amgen, AstraZeneca, Bayer, JNJ, and Novartis. Shahneen Sandhu reports consulting/advisory roles with Abbvie, AstraZeneca, Bristol-Myers Squibb, Merck Sharp & Dohme, Novartis, Roche/Genentech, Janssen, AdvanCell, Daiichi Sankyo, Synolo Therapeutics, Macrogenics, ERASCA and Skyline Diagnostics; honoraria to the institution from Abbvie, AstraZeneca, Bristol-Myers Squibb, Janssen, Merck, Merck Serono, Novartis, AdvanCell, and Skyline Dx; institutional research funding from Amgen, AstraZeneca, Bristol-Myers Squibb, Endocyte (a Novartis company), Genentech/Roche, Merck, Novartis, Pfizer, and Senhwa Biosciences; and stock ownership in AdvanCell. Louise Emmett receives consultant fees from Advanced Accelerator Applications, a Novartis company, Astellas, Clarity Pharma, and Advancell; receives research grants from Advanced Accelerator Applications, a Novartis company, and Clarity Pharma; and has stock options or other ownership interests with AdvanCell and Clarity Pharma. Clemens Kratochwil is a coinventor of patents in the field of PSMA-targeted radiopharmaceutical therapy; receives consulting fees from AAA/Novartis, Endocyte, Bayer, Roche, AdvanCell, Telix, and SOFIE Biosciences; has stock options or other ownership interest with FAPI-Holding AG and AdvanCell; and receives nonfinancial support from ABX and ABX-CRO. No other potential conflict of interest relevant to this article was reported.
